# Which unmet social care needs have the biggest impact on healthy ageing? An analysis of data from the English Longitudinal Study of Ageing

**DOI:** 10.1136/bmjopen-2024-084812

**Published:** 2025-01-22

**Authors:** Gemma F Spiers, Laurie E Davies, David Sinclair, Michelle M C Tan, Andrew Kingston, Barbara Hanratty

**Affiliations:** 1 Newcastle University, Newcastle upon Tyne, UK

**Keywords:** Aged, Health Equity, Health Services for the Aged

## Abstract

**Abstract:**

**Background and aim:**

Unmet need for social care is linked to numerous adverse health outcomes. Understanding which unmet needs have the biggest impact on healthy ageing could help resource-stretched services prioritise care. To address this evidence gap, our analysis aimed to explore the association between selected individual unmet care needs and an indicator of healthy ageing.

**Design and data:**

Cross-sectional analysis of data from the English Longitudinal Study of Ageing (Wave 9). A total of 6109 people aged 50 years or over, with complete data items, formed the basis for this analysis.

**Measures:**

Absolute unmet need for help with each: walking 100 yards and climbing one flight of steps (mobility); managing money, managing medication, doing housework and shopping for groceries (instrumental activities of daily living (IADLs)); and dressing, walking across a room, bathing or showering, eating, using the toilet and getting in and out of bed (activities of daily living (ADLs)). Our outcome measure was poor self-rated health .

**Results:**

Associations between poor self-rated health and most unmet ADL, IADL and mobility needs were not statistically significant. People with an unmet need for support with managing money were nine times more likely to report poor self-rated health than those whose support needs were met in this domain (OR=9.23, 95% CI: 2.12 to 40.23). In a comparison of people with met and unmet needs, individuals with met needs had higher levels of dependency than those with unmet needs.

**Conclusions:**

Some unmet needs may be especially consequential for older people’s health. However, shortcomings in current data limit a clear and confident assessment of this. Our analysis highlights the importance of data on the level of need to better understand the link between unmet care needs and healthy ageing.

**STRENGTHS AND LIMITATIONS OF THIS STUDY**

- Our analysis drew on contemporary data collected in 2019 using a representative sample.
- We adjusted for key confounders, including age, sex, wealth and disease count.
- Heterogeneity in the severity of difficulty with each activities of daily living (ADL), instrumental ADL and mobility activity undermined our attempt to compare health outcomes between populations with met and unmet need for these activities.
- Our work highlights the need for more robust data on unmet social care needs to understand the impact on healthy ageing.

## Background

 An unmet social care need arises when people could benefit from help with their daily lives but do not receive support. Such needs may include, for example, help with activities of daily living (ADLs), instrumental ADL (IADLs) and mobility. Needs may be considered unmet when no help is received (absolute unmet need) or when help received is judged to be insufficient or inadequate (relative unmet need).^1^ Recent estimates suggest that in England, 1.6 million adults aged 65+ years have an absolute or relative unmet need for social care support with at least one ADL.^2^ Some groups are at higher risk of having unmet social care needs, such as populations on lower incomes.^3^

Unmet social care needs are linked to poor health outcomes. Discomfort, weight loss, dehydration, falls, burns,^4^ a greater risk of using emergency care (particularly for falls and injuries),^5^ an increased risk of hospital admission and readmission^6 7^ and an increased risk of mortality^8^ are all observed consequences of unmet need for help with ADLs, IADLs and mobility limitations. This evidence is not surprising: ADLs, IADLs and mobility are underpinned by physical activity, cognitive stimulation and social interactions, all of which are integral to healthy ageing.^9^,^11^ A lack of support with such needs will thus inevitably lead to a decline in health. This is likely to be a bidirectional relationship, with poor health further compromising older people’s ability to maintain independence with day-to-day tasks.

Although unmet need for support with ADLs, IADLs and mobility are linked to poor health, our understanding of this remains cursory. Previous research has typically explored unmet need as a composite of multiple areas of need. The range of possible care needs that may go unmet is diverse, spanning several ADLs, IADLs and mobility difficulties. Feasibly, all difficulties with ADLs, IADLs and mobility, which are not countered with support, are likely to have an adverse impact on the health of older people. However, no research to date has explored which of these individual unmet ADL, IADL and mobility support needs have the biggest impact on healthy ageing. This is important as it may not be realistic for all needs to be addressed by resource-stretched care services. However, if certain needs have a bigger adverse impact on health than others, there may be an argument to prioritise and target such needs in policy and practice.

To address this gap in evidence, we aimed to explore the association between individual unmet ADL, IADL and mobility needs and healthy ageing.

## Methods

We undertook a cross-sectional analysis of data from the English Longitudinal Study of Ageing (ELSA), an ongoing nationally representative study of adults aged 50 years and over in England.^12^ ELSA is a sister study of the US Health and Retirement Study.^13^ Participants are recruited from households in England but continue to be surveyed if they move into a care home. Refreshment samples are added periodically to maintain population representativeness. Full details of the recruitment and survey methods for ELSA are reported elsewhere.^14^

### Population

Using data from the most recent study wave (Wave 9, 2019), 6109 (unweighted) people aged 50 or over, with complete data items, formed the basis for this analysis. With weighting, the final sample was 6136 participants.

### Measures: exposure, outcome and covariates

A measure of absolute unmet need was created for each self-reported ADL, IADL and mobility need available in ELSA. Absolute unmet need describes the number of people who have difficulty, but receive no help, with an ADL/IADL/mobility limitation.^1^ This operationalisation of unmet need was used because data are not available in ELSA to quantify the relative unmet need for individual ADL, IADL and mobility need items.

The measures of individual unmet need were created in two steps. First, we selected the needs relevant to this analysis. These were self-reported difficulties with walking 100 yards and climbing one flight of steps (mobility); managing money, managing medication, doing housework and shopping for groceries (IADLs); and dressing, walking across a room, bathing or showering, eating, using the toilet and getting in and out of bed (ADLs). These 12 needs were selected because ELSA recorded whether help was received for them, which enabled us to quantify unmet need. Another mobility variable (difficulty climbing several flights of steps) was available but not selected due to the risk of collinearity with the included variable, difficulty climbing one flight of steps.

Second, for each need, we determined whether help was received and used this to create three response categories: (1) no self-reported difficulty (no need), (2) self-reported difficulty and receives help from a person (met need) and (3) self-reported difficulty and no help from a person (unmet need). We included a ‘no need’ category in our measure of unmet need. This allowed us to include all participants in the model, whether or not they reported difficulty with all ADL, IADL and mobility limitations (see Analysis, below).

To examine the association between the individual unmet needs and healthy ageing, we used self-rated health as our outcome measure. Self-rated health was selected as it is a strong indicator of health and mortality in older populations.^15^ A binary version of this variable was created to differentiate populations with poor self-rated health and excellent/very good/good/fair self-rated health.

Covariates selected for this analysis included age, sex, total net non-pension wealth as an indicator of socioeconomic status and disease count. Total net non-pension wealth was selected as this is a robust indicator of socioeconomic status in older populations.^16 17^ This measure combines net housing wealth, net non-housing wealth and net financial wealth. In ELSA, this measure is available as quintiles. For this analysis, we merged quintiles 2–4 to create three categories: low, medium and high wealth. Disease count was used as an indicator of multimorbidity: diseases were selected based on those used in previous research.^18^

### Analysis

Logistic regression was used to model the association between all twelve individual unmet needs and the outcome of poor self-rated health. This model was adjusted for age, sex, total net non-pension wealth and disease count. In a post-hoc analysis, we then compared the health profiles of the study populations with met and unmet needs, in terms of disease count and use of aids and adaptations. We did this to test our hypothesis that individuals with *met* needs—who were more likely to report poor self-rated health—had a greater level of dependency than those with unmet needs. All analyses were weighted by the cross-sectional weight using the survey package^19^ in R V.3.6.0.

### Patient and public involvement

There was no patient and public involvement in this study. 

## Results

Table 1 summarises the characteristics of the study population. Just over half of participants were female (51.4%). Almost half of participants reported two or more long-term conditions (46.6%). Difficulties with ADLs, IADLs and mobility were rare: for most needs, difficulties were reported by fewer than 10% of participants. The most common unmet needs were climbing one flight of stairs (8.6%), walking 100 yards (6.4%) and dressing (6.2%).

**Table 1:** Characteristics of the study sample (weighted n=6136)

Characteristic		Percentage (count)
Sex	Male	48.6 (2983)
	Female	51.4 (3153)
Age group (years)	50–59	36.6 (2243)
	60–69	30.5 (1870)
	70–79	22.1 (1354)
	80+	10.9 (668)
Wealth group	Poorest group	18.5 (1136)
	Middle group	60.8 (3733)
	Richest group	20.6 (1267)
Disease groups	0	21.4 (1312)
	1	32.0 (1962)
	2+	46.6 (2862)
Self-rated health	Poor	6.1 (372)
	Excellent/very good/good/fair	93.9 (5763)
Need: managing money	No needs	97.8 (5999)
	Met need	1.7 (105)
	Unmet need	0.5 (32)
Need: taking medication	No needs	98.3 (6030)
	Met need	1.2 (71)
	Unmet need	0.6 (35)
Need: walking 100 yards	No needs	91.7 (5627)
	Met need	1.8 (113)
	Unmet need	6.4 (396)
Need: walking across a room	No needs	97.9 (6005)
	Met need	0.5 (30)
	Unmet need	1.7 (101)
Need: getting in/out of bed	No needs	96.0 (5888)
	Met need	0.7 (46)
	Unmet need	3.3 (202)
Need: climbing one flight of steps	No needs	90.4 (5546)
	Met need	1.0 (63)
	Unmet need	8.6 (526)
Need: bathing or showering	No needs	94.3 (5784)
	Met need	2.2 (133)
	Unmet need	3.6 (219)
Need: using the toilet	No needs	97.5 (5983)
	Met need	0.4 (25)
	Unmet need	2.1 (127)
Need: eating	No needs	98.5 (6045)
	Met need	0.7 (45)
	Unmet need	0.7 (45)
Need: work around house/garden	No needs	89.4 (5486)
	Met need	6.6 (405)
	Unmet need	4.0 (244)
Need: dressing	No needs	90.7 (5564)
	Met need	3.1 (192)
	Unmet need	6.2 (379)
Need: shopping	No needs	93.7 (5749)
	Met need	5.1 (311)
	Unmet need	1.2 (75)

Numbers are weighted and rounded.

Table 2 presents the model exploring the association between the 12 unmet needs and poor self-rated health, adjusting for covariates. Compared with people with met needs, those with unmet need for support (with managing money, managing medication, getting in and out of bed, bathing and showering and shopping) were more likely to report poor self-rated health. Only one of these associations was statistically significant at the 95% CI (managing money, OR=9.23, 95% CI 2.12 to 40.23, p<0.01). For the remaining need variables, people with unmet needs were less likely to be in poor health compared with those whose needs were met on these measures. However, these associations were not statistically significant at the 95% CI.

**Table 2:** OR of poor self-rated health for populations with unmet need or no need; met need is the referent

Self-reported difficulties	No need	Unmet need
Managing money	1.59 (0.66–3.84), p=0.31	9.23 (2.12–40.23), p<0.01
Managing medication	0.78 (0.26–2.34), p=0.66	4.14 (0.78–21.98), p=0.10
Walking 100 yards	0.23 (0.11–0.50), p<0.001	0.73 (0.36–1.47), p=0.38
Walking across a room	0.73 (0.20–2.62), p=0.63	0.68 (0.19–2.38), p=0.54
Getting in/out of bed	1.51 (0.49–4.69), p=0.47	2.82 (0.88–9.01), p=0.08
Climbing one flight of steps	0.54 (0.20–1.46), p=0.22	0.90 (0.35–2.28), p=0.82
Bathing and showering	0.67 (0.32–1.42), p=0.30	1.21 (0.55–2.66), p=0.64
Using the toilet	1.19 (0.27–5.17), p=0.82	0.73 (0.16–3.32), p=0.68
Eating	1.01 (0.26–3.96), p=0.99	0.64 (0.12–3.29), p=0.59
Gardening and housework	0.22 (0.12–0.39), p<0.001	0.67 (0.36–1.26), p=0.22
Dressing	0.60 (0.30–1.18), p=0.14	0.81 (0.40–1.63), p=0.56
Shopping	1.11 (0.58–2.11), p=0.75	1.18 (0.46–3.00), p=0.73

Model adjusted for age, sex, disease count, total net non-pension wealth, and all needs, with met need as the referent.

Contrary to previous evidence, most unmet ADL, IADL and mobility needs were not clearly linked with poor health in these data. To further explore these findings, we hypothesised that the subgroups with met and unmet need in our analysis did not have equivalent levels of help needed for each ADL, IADL and mobility limitation. If people with the greatest need for help with these limitations are the most likely to access support that may explain why people with met needs were more likely to be in poor health compared with those with unmet needs.

To explore this, we compared the health profiles for the subgroups with met and unmet need. Overall, participants with met needs used more aids and adaptations than those with unmet needs (online supplemental materials table A). Multimorbidity presents a more complex picture (online supplemental materials table B). For some ADLs, IADLs and mobility difficulties, the proportions of people with met and unmet needs were similar across levels of multimorbidity. For other difficulties, the figures indicated that people living with a higher number of conditions were more likely to have met needs.

This analysis supports our hypothesis that individuals with met needs had a greater level of dependency than those with unmet needs.

## Discussion

This analysis does not support a clear link between unmet needs and poor health, contrary to previous research.^4^,^8^

Poor health was more likely among older people with unmet needs for support with managing money, managing medication, getting in and out of bed, bathing and showering and shopping. Only one of these associations was statistically significant: unmet need for help managing money. This suggests that support for managing money may be especially important for older people’s health. This is reasonable, as difficulties managing money will limit many other crucial aspects of day-to-day life, such as travelling to and from appointments, buying food and heating homes. However, an unmet need for support with managing money is unlikely to be the only unmet need implicated in healthy ageing.

An important consideration to these findings concerns the nature of the data. Our findings may stem from the use of binary disability variables in ELSA. That is, participants respond yes or no when asked if they have difficulty with a particular ADL, IADL or mobility activity. Such a binary response provides no information on the level of severity of difficulty. This means that study participants reporting difficulty with an activity may be heterogeneous in their need for support with that activity. When we added information on whether help was received, in order to define unmet need (eg, does anyone help you with this activity, yes or no), this appeared to have had the unintended effect of differentiating people with dependency from those with disability. This explanation is supported by the greater use of aids and adaptations among those with met needs in our study population.

A second explanation is that isolated unmet ADL, IADL and mobility needs may be less consequential for health than a combination of unmet needs. Specifically, when certain needs go unmet, this is likely to impact a person’s ability to carry out other ADLs and IADLs. Any interaction between multiple unmet needs would not be accounted for in our attempt to quantify the independent contributions of individual unmet needs to poor health.

Our analysis also made use of cross-sectional data. This approach may not be ideal to examine the potential impact of individual unmet care needs on health, especially where such impact may manifest over time. Longitudinal studies of health trajectories and changing support for daily living may offer greater insights, as well as confirm temporality between unmet needs and poor health.

Finally, the statistical significance of the unmet need for managing money variable may not reflect any meaningful link between having no support for this IADL and poor health. Rather, this may reflect a possible misinterpretation of the survey question for this activity. In ELSA, participants are asked if they have any difficulties with managing money such as paying bills or expenses, due to a physical, mental, emotional or memory problem.^20^ It is possible that this question is (mis)interpreted as experiencing financial difficulties or pressures, rather than experiencing difficulties because of the cognitive and organisational demands of the activity.

In summary, we would expect that most, if not all, of these unmet needs to have consequences for healthy ageing. However, more granular data is needed to confidently understand the independent impact of each type of unmet need on older people’s health.

### Strengths and limitations

Unmet needs are an important part of understanding healthy ageing, especially when care provision is not universal. Previous evidence also highlights the impact of unmet needs on health, and the link between the two is not without a rational foundation. Our attempt to understand the independent contribution of *individual* unmet needs to heathy ageing was novel but challenged by limitations in our dataset.

Our analysis makes use of contemporary data collected in 2019 using a representative sample. We adjusted for key confounders, including age, sex, wealth and disease count. Changes in the estimates between iterations of the model indicated a risk of the model being overfitted, thus potentially reducing the reliability of the findings. We chose therefore not to explore any additional confounding variables in the analysis. While this is a limitation of the study, it is not a major shortcoming given the other, more critical limitations of the data described earlier.

The ELSA study population is generally considered to be representative of the over 50 population in England. Survey designs often oversample more advantaged populations, which risks underestimations of poor health and disability. However, we applied established weights to the data to overcome potential bias relating to an underrepresented study sample. The data also represent a pre-COVID-19 population, and it is therefore possible that the link between unmet social care needs may look different in post-COVID-19 data given the prolonged social isolation experienced by older people during the pandemic.

Finally, the proportion of participants reporting difficulties with each ADL, IADL and mobility limitation (with either met or unmet need) was small. This is not uncommon and mirrors similarly small proportions of participants reporting ADL, IADL and mobility difficulties in other studies.^21 22^ Such small proportions may partly account for the imprecision in CIs observed in this analysis, which limits the conclusions we can draw.

### Implications for research

Quantifying unmet care needs is complex. Different ways of identifying need (perceived or assumed), the support received or not (paid, unpaid, both) and whether such need is unmet (absolute, relative) highlight the myriad of ways the concept can be operationalised.

Our analysis points to another key challenge: a binary measure of disability may group together people with heterogeneous needs for support. Satisfying an assumption of equivalence in need is important as it allows a fair comparison of the health equity consequences when some people do not receive support.

Going forward, there is a need to optimise nationally representative data to be able to answer questions about healthy ageing and unmet care needs. Specifically, this could include data that identifies the degree of difficulties with activities, perceived need for help and perceived unmet need and detail of the quantity and quality of any support received.

## Conclusion

Understanding which unmet ADL, IADL and mobility needs have the biggest impact on healthy ageing is important. Some unmet needs (managing money, managing medication, getting in and out of bed, bathing and showering, and shopping) may be especially consequential for older people’s health. However, shortcomings to current data limit a clear and confident assessment of this. Our analysis highlights the importance of data on the level of need to better understand the link between unmet care needs and healthy ageing.

## supplementary material

10.1136/bmjopen-2024-084812 online supplemental file 1

## Data Availability

Data sharing is not applicable as no datasets were generated and/or analysed for this study.
