# Supplementary material for: Which unmet social care needs have the biggest impact on healthy ageing? An analysis of data from the English Longitudinal Study of Ageing
Source: BMJ Open. 2025 Jan 22;15(1):e084812. doi: 10.1136/bmjopen-2024-084812 (PMC11758690; doi:10.1136/bmjopen-2024-084812)
Supplement: online supplemental file 1 [file bmjopen-15-1-s001.docx]

Supplementary materials

**Table A. Proportion of participants using each number of aids and adaptations by need response, for each ADL, IADL and mobility difficulty**

| **ADL/IADL/Mobility limitation and category of need** | | **Number of aids/adaptations, prevalence (%)** | | | |
| --- | --- | --- | --- | --- | --- |
|  |  | *0* | *1* | *2* | *3+* |
| Managing money | No need  Met  Unmet | 88.4  20.4  65.4 | 8.0  28.6  30.6 | 2.3  28.7  0.0 | 1.2  22.3  4.0 |
| Taking medication | No need  Met  Unmet | 88.3  17.7  31.6 | 8.1  29.5  27.2 | 2.4  25.4  22.0 | 1.2  27.5  19.2 |
| Walking 100 yards | No need  Met  Unmet | 92.8  8.9  28.4 | 6.0  31.7  38.0 | 0.9  34.7  20.1 | 0.3  24.7  13.5 |
| Walking across a room | No need  Met  Unmet | 88.8  2.7  9.8 | 8.1  27.0  27.0 | 2.1  38.9  30.1 | 0.9  31.4  33.0 |
| Getting in/out of bed | No need  Met  Unmet | 89.3  9.9  39.6 | 7.6  28.4  30.3 | 2.0  39.1  15.5 | 1.0  22.5  14.6 |
| Climbing one flight of steps | No need  Met  Unmet | 92.8  11.4  36.3 | 5.9  41.9  32.1 | 1.0  27.7  18.1 | 0.3  19.1  13.5 |
| Bathing or showering | No need  Met  Unmet | 90.7  10.8  38.9 | 7.0  35.2  33.1 | 1.7  27.3  16.0 | 0.6  26.7  12.1 |
| Using the toilet | No need  Met  Unmet | 88.7  0.0  30.2 | 7.9  18.7  33.2 | 2.2  53.7  19.0 | 1.2  27.6  17.6 |
| Eating | No need  Met  Unmet | 87.9  19.3  46.0 | 8.3  23.7  16.0 | 2.4  33.1  25.4 | 1.4  23.9  12.7 |
| Doing work around house/garden | No need  Met  Unmet | 93.4  23.6  51.6 | 5.5  34.0  33.5 | 0.8  25.9  8.5 | 0.3  16.6  6.4 |
| Dressing | No need  Met  Unmet | 91.6  22.2  54.3 | 6.2  35.1  28.5 | 1.5  25.3  10.6 | 0.7  17.4  6.7 |
| Shopping | No need  Met  Unmet | 91.2  22.7  39.4 | 7.0  29.4  35.7 | 1.2  28.5  14.1 | 0.5  19.4  10.8 |

*Rows may not sum to 100% due to rounding.*

**Table B. Proportion of participants with each number of long-term conditions by need response, for each ADL, IADL and mobility need**

|  | | **Number of long-term conditions (%)** | | | | | | | |
| --- | --- | --- | --- | --- | --- | --- | --- | --- | --- |
|  |  | **0** | **1** | **2** | **3** | **4** | **5** | **6** | **7** |
| Managing money | No need  Met  Unmet | 21.9  0.7  2.8 | 32.4  14.2  12.3 | 24.7  20.9  23.9 | 13.9  24.4  48.3 | 5.4  26.6  5.2 | 1.6  6.4  3.6 | 0.2  5.6  0.0 | 0.0  1.1  4.0 |
| Managing medication | No need  Met  Unmet | 21.8  1.0  0.0 | 32.4  9.7  6.7 | 24.7  24.7  6.1 | 14.0  22.5  46.1 | 5.4  24.9  27.8 | 1.5  10.5  2.4 | 0.2  6.6  5.2 | 0.0  0.0  5.8 |
| Walking 100 yards | No need  Met  Unmet | 23.1  0.6  3.2 | 34.0  6.0  10.7 | 24.6  22.1  25.8 | 12.9  30.0  29.3 | 4.4  25.1  19.5 | 1.0  10.8  8.9 | 0.1  3.1  2.2 | 0.0  2.2  0.5 |
| Walking across room | No need  Met  Unmet | 21.8  5.5  0.0 | 32.5  5.4  8.2 | 24.6  26.5  26.2 | 13.9  13.5  33.7 | 5.4  35.6  16.4 | 1.5  9.5  7.4 | 0.2  5.1  4.0 | 0.0  0.0  3.1 |
| Getting in and out of bed | No need  Met  Unmet | 22.2  3.6  1.2 | 33.0  3.7  9.2 | 24.5  26.4  28.8 | 13.7  28.2  27.3 | 5.0  24.9  22.5 | 1.4  9.1  7.1 | 0.2  4.1  2.4 | 0.0  0.0  1.6 |
| Climbing one flight of steps | No need  Met  Unmet | 23.3  3.5  3.7 | 34.4  0.8  9.7 | 24.6  28.6  24.5 | 12.7  29.1  28.7 | 3.9  22.6  23.1 | 1.0  12.3  7.5 | 0.1  1.1  2.1 | 0.0  1.9  0.6 |
| Bathing and showering | No need  Met  Unmet | 22.5  1.1  4.8 | 33.3  7.2  11.3 | 24.7  26.1  22.7 | 13.5  22.3  28.6 | 4.7  26.5  21.2 | 1.1  11.7  10.2 | 0.2  1.8  1.2 | 0.0  3.3  0.0 |
| Using the toilet | No need  Met  Unmet | 21.9  3.5  3.3 | 32.6  4.1  9.1 | 24.7  26.7  20.9 | 13.9  26.0  29.3 | 5.4  23.8  20.3 | 1.4  11.1  12.3 | 0.2  4.7  1.4 | 0.0  0.0  3.5 |
| Eating | No need  Met  Unmet | 21.7  0.0  3.9 | 32.3  7.9  7.4 | 24.7  22.6  16.3 | 14.0  21.4  35.4 | 5.4  38.1  20.8 | 1.5  4.4  13.5 | 0.2  2.7  2.6 | 0.1  2.8  0.0 |
| Housework and gardening | No need  Met  Unmet | 23.5  2.3  4.8 | 34.4  10.7  13.0 | 24.6  22.1  29.2 | 12.5  28.2  30.3 | 3.9  24.4  15.9 | 0.9  8.9  5.4 | 0.1  2.7  0.9 | 0.0  0.8  0.5 |
| Dressing | No need  Met  Unmet | 23.1  1.2  6.0 | 33.9  9.2  14.7 | 24.0  27.0  27.1 | 12.9  30.3  26.7 | 4.4  22.5  16.7 | 1.1  6.7  7.9 | 0.2  1.5  0.7 | 0.0  1.7  0.3 |
| Shopping | No need  Met  Unmet | 22.7  2.5  0.0 | 33.6  9.4  4.1 | 24.7  23.8  20.6 | 13.2  28.3  38.0 | 4.6  22.8  19.1 | 1.1  9.4  13.4 | 0.1  2.6  3.8 | 0.0  1.2  1.0 |

*Rows may not sum to 100% due to rounding*.
