# Supplementary material for: Which unmet social care needs have the biggest impact on healthy ageing? An analysis of data from the English Longitudinal Study of Ageing
Source: BMJ Open. 2025 Jan 22;15(1):e084812. doi: 10.1136/bmjopen-2024-084812 (PMC11758690; doi:10.1136/bmjopen-2024-084812)
Supplement: Supplementary file 2 [file bmjopen-2024-084812.reviewer_comments.pdf]

PEER REVIEW HISTORY

BMJ Open publishes all reviews undertaken for accepted manuscripts. Reviewers are asked to complete a checklist review form and are provided with free text boxes to elaborate on their assessment. These free text comments are reproduced below.

ARTICLE DETAILS

Title (Provisional)

Which unmet social care needs have the biggest impact on healthy ageing? An analysis of data from the English Longitudinal Study of Ageing

Authors

Spiers, Gemma F; Davies, Laurie E.; Sinclair , David; Tan, Michelle MC; Kingston, Andrew; Hanratty, Barbara

---

VERSION 1 - REVIEW

**Reviewer**	**1**
**Name**	**Chakraborty, Amal**
**Affiliation**	**The University of Sydney, Faculty of Medicine and Health**
**Date**	**23-Feb-2024**
**COI**	**None**

Thank you for the opportunity to review this article. The authors have tackled and reported an important topic of research in the manuscript. It’s an interesting study.

I just have a couple of minor comments for authors to address:

- add the corresponding p values for each measure in Table 2

- also add the p value on line 27 to the sentence: "Only one of these associations was statistically significant at the 95% confidence interval (managing money, OR=9.23, 95% CI 2.12-40.23)."

**Reviewer**	**2**
**Name**	**Attisso, Eugene**
**Affiliation**	**Quebec National Institute of Public Health**
**Date**	**07-Apr-2024**

COI **No conflits of interests**

Introduction

Comment 1: At page 4 Line 5-6, You define unmet need care with no reference. Please provide it. Otherwise, I'm not sure these sentences were correctly formulated. Because, you focus the concept of unmet need care on ADLs. But, the unmet care needs included all healthcare needs (diagnostics, treatment, long-term care etc.). These cares need can also be an assistance or help for **inabilities** of ADLs. I make important consideration for the word **inability or lost of ADLs**. Because, the care needs will be required when the people have an **inability or loss** of at least one ADLs. Ref: Academy of Medical Sciences. Unmet need in healthcare: summary of a roundtable held at the Academy of Medical Sciences on 31 July 2017, held with support from the British Academy and NHS England. 2017. https://acmedsci.ac.uk/file-download/430378

Furthermore, I'm not sure too that ADLs was different from IADLs and mobility. ADLs (Activities of Daily living) were an indication of human's functional status. There are two type of ADLs: basic ADLs and instrumental ADLs. The basic activities of daily living (BADLs) or physical ADLs are those skills required to manage one's basic physical needs (mobility, feeding, dressing, personal hygiene and Toileting). The Instrumental Activities of Daily Living (IADLs) include more complex activities related to the ability to live independently in the community. This would include activities such as managing finances and medications, food preparation, housekeeping, and laundry. https://www-ncbi-nlm-nih-gov.acces.bibl.ulaval.ca/books/NBK470404/

Comment 2: In the second paragraph, it's important to clarify the causal pathway between ageing, loss of ADLs and health status. You can mention also the "chicken and egg situation" between the loss of health status, the loss of ADLs and the unmet social support care. Indeed, the ageing was one of cause of ADLs lost. It was also the cause of poor health status due to chronic disease and mental health disorders etc. This poor health status can also cause the loss of ADLs. The unmet social support care can also cause a poor health status, and vice versa. Otherwise individuals, societal and healthcare system organisation factors can also cause unmet social support care needs.

Good demonstration of the gaps and scope of this work!

Methods

The common definition of elders is people aged 65 years and over. Why, you choose 50 years and over?

It's secondary data, thus it's important to explain how these data were collected. It's also important to mention the participation rate of the cycle 9. Otherwise, I don't see the flow chart of participants selection (according to inclusion and exclusion criterions).

Please provide citation beside the ELSA instrument used to measure ADLs components.

You must use theses subheading in our methods: database, population or sample, exposure, outcomes and co-variables.

This will be useful to include in your analysis the variable about the level of schooling, ethnicity and residency (rural or urban) of our participants.

Results

It may be useful to report that “climbing”, walking and dressing were the most unmet social care among all ADLs.

The post hoc analysis reported in second and third paragraph must be specified in method section.

Discussion

The first paragraph the discussion must report the summary of the results according the STROBE checklist. I don’t see it!

You blend the discussion; for example, your current strengths and limits section weren’t! Please remake it according to strobe guidelines.

Some limits were important to be mentioned

1) Selection bias: participation rate bias due to survey design and his effect on the measure of association. Otherwise, his effect on representativeness of our study sample.
2) Information bias: you must mention that the self reported measure of variables can lead under or over estimate of the real status of participants because of memory bias or social desirability bias. Theses situations can be differential because people with poor health can remember or attributed it to the ADLs incapacities.
3) Confounding bias: others factors like level of schooling, ethnicity, residency, social support accessibility or healthcare accessibility, environmental factors (weather, road, light, housing etc..) were not take in account
4) This is a cross sectional study, there isn’t temporality between exposure and outcomes. We can’t conclude that poor health was presence before or after the unmet financial managing support.

The small size of people exposed to unmet need for social support care can’t also explained the large CI95% around the ORs so the imprecision of these estimates.

**Reviewer**	**3**
**Name**	**Aggar, Christina**
**Affiliation**	**Southern Cross University, Faculty of Health**
**Date**	**07-Aug-2024**
**COI**	**nil**

It is an interesting paper, however the data was pre COVID and I think that this needs to be acknowledged. We now know that loneliness is one of the most important social care needs that impact healthy ageing. Another question I would ask - who supported the unmet need/s, family or formal carers, as this is also known to impact healthy ageing. Otherwise the shortcomings of the current data, whilst many, have been addressed

---

VERSION 1 - AUTHOR RESPONSE

Reviewer comment	Author response
Reviewer: 1<br>Dr. Amal Chakraborty, The University of Sydney<br>Comments to the Author:<br>Thank you for the opportunity to review this article. The authors have tackled and reported an important topic of research in the manuscript. It's an interesting study.<br><br>I just have a couple of minor comments for authors to address:<br>- add the corresponding p values for each measure in Table 2<br>- also add the p value on line 27 to the sentence: "Only one of these associations was statistically significant at the 95% confidence interval (managing money, OR=9.23, 95% CI 2.12-40.23)."	Thank you for these helpful suggestions. We have added p values to Table 2 and to the sentence suggested.
Reviewer: 2<br>Dr. Eugene Attisso, Quebec National Institute of Public Health<br>Comments to the Author:<br>Introduction<br>Comment 1: At page 4 Line 5-6, You define unmet need care with no reference. Please provide it. Otherwise, I'm not sure these sentences were correctly formulated. Because, you focus the concept of unmet need care on ADLs. But, the unmet care needs included all healthcare needs (diagnostics, treatment, long-term care etc.). These cares need can also be an assistance or help for inabilities of ADLs. I make important consideration for the word inability or lost of ADLs. Because, the care needs will be required when the people have an inability or loss of at least one ADLs. Ref: Academy of Medical Sciences. Unmet need in healthcare: summary of a roundtable held at the Academy of Medical Sciences on 31 July 2017, held with support from the British Academy and NHS England. 2017. https://acmedsci.ac.uk/file-download/430378<br>Furthermore, I'm not sure too that ADLs was different from IADLs and mobility. ADLs (Activities of Daily living) were an indication of human's functional status. There are two type of ADLs: basic ADLs and instrumental ADLs. The	Thank you: on our version of the submitted manuscript, there is in fact a citation (Vlachantoni 2019) for the definition of unmet need (where we describe both relative and absolute).<br><br>You posit that unmet care needs can include all healthcare needs. We would agree that the term 'unmet needs' can encompass a wide range of health and social care needs. However, in this work, our focus was on unmet social care needs. To clarify this, we have amended the very first sentence to indicate the focus is on unmet social care needs. Elsewhere in the paper it is explicit that the focus is on social care (ADL, IADL, mobility) needs, including the manuscript title.

basic activities of daily living (BADLs) or physical ADLs are those skills required to manage one's basic physical needs (mobility, feeding, dressing, personal hygiene and Toileting). The Instrumental Activities of Daily Living (IADLs) include more complex activities related to the ability to live independently in the community. This would include activities such as managing finances and medications, food preparation, housekeeping, and laundry. https://www-ncbi-nlm-nih-gov.acces.bibl.ulaval.ca/books/NBK470404/	
Comment 2: In the second paragraph, it's important to clarify the causal pathway between ageing, loss of ADLs and health status. You can mention also the "chicken and egg situation" between the loss of health status, the loss of ADLs and the unmet social support care. Indeed, the ageing was one of cause of ADLs lost. It was also the cause of poor health status due to chronic disease and mental health disorders etc. This poor health status can also cause the loss of ADLs. The unmet social support care can also cause a poor health status, and vice versa. Otherwise individuals, societal and healthcare system organisation factors can also cause unmet social support care needs.<br>Good demonstration of the gaps and scope of this work!	Thank you – we agree this would be a helpful addition to the second paragraph where we already describe this causal pathway. We have added the following:<br><br>This is likely to be a bidirectional relationship, with poor health further compromising older people's ability to maintain independence with day-to-day tasks.
Methods<br>The common definition of elders is people aged 65 years and over. Why, you choose 50 years and over?	Thank you for the opportunity to clarify this. Whilst there is no agreed consensus on the definition on older populations, we chose to use a study population aged 50 years and over to account for the fact that age related poor health occurs earlier in the lifecourse for the most disadvantaged populations. Limiting the sample to populations aged 65 and over would omit disadvantaged populations who are experiencing significant functional limitations earlier in life.
It's secondary data, thus it's important to explain how these data were collected. It's also important to mention the participation rate of the cycle 9. Otherwise, I don't see the flow chart of participants selection (according to inclusion and exclusion criterions).	Thank you for highlighting this. The full details for recruitment to ELSA and participation rates are reported elsewhere. We have added further details (and linked citation) to the methods outlining ELSA's recruitment process:<br>We undertook a cross-sectional analysis of data from the English Longitudinal Study of Ageing (ELSA), an ongoing nationally representative study of adults aged 50 and

	over in England.[12] It is a sister-study of the US Health and Retirement Study (HRS). Participants are recruited from households in England but continue to be surveyed if they move into a care home. Refreshment samples are added periodically to maintain population representativeness. Full details of the recruitment and survey methods for ELSA are reported elsewhere.[13]
Please provide citation beside the ELSA instrument used to measure ADLs components	There is no citation to give for these measures as they are simply self-reported difficulties with the ADLs, IADs and mobility activities listed. However, we have added a citation for measuring absolute unmet needs.
.<br>You must use theses subheading in our methods: database, population or sample, exposure, outcomes and co-variables.<br>This will be useful to include in your analysis the variable about the level of schooling, ethnicity and residency (rural or urban) of our participants.	We have amended some of these subheadings to reflect this suggestion. However, we have omitted data source subheading as this is explicit within the opening paragraph of the methods.
Results<br>It may be useful to report that "climbing", walking and dressing were the most unmet social care among all ADLs.	Thank you for this suggestion – we have added a sentence to the results to describe this.
The post hoc analysis reported in second and third paragraph must be specified in method section.	Thank you for your comment. We have made the post-hoc analysis more explicit in the methods section.
Discussion<br>The first paragraph the discussion must report the summary of the results according the STROBE checklist. I don't see it!<br>You blend the discussion; for example, your current strengths and limits section weren't! Please remake it according to strobe guidelines.	Thank you for referring to the STROBE checklist, which we included in our manuscript submission. We have included the advised contents of the STROBE checklist in our discussion (key findings, interpretation, limitations, generalisability), but without these explicit headings. This is because we used the headings advised by the journal guidelines. However, if the editorial team would prefer explicit STROBE headings, we can add these in.
Some limits were important to be mentioned<br>1) Selection bias: participation rate bias due to survey design and his effect on the measure of association. Otherwise, his effect on representativeness of our study sample.	Thank you – we have added the following to the discussion:<br><br>The ELSA study population is generally considered to be representative of the over 50 population in England. Survey designs often oversample more advantaged populations, which risks underestimations

	of poor health and disability. However, we applied established weights to the data to overcome potential bias relating an underrepresented study sample. The data also represent a pre-COVID population, and it is therefore possible that the link between unmet social care needs may look different in post-COVID data given the prolonged social isolation experienced by older people during the pandemic.
2) Information bias: you must mention that the self reported measure of variables can lead under or over estimate of the real status of participants because of memory bias or social desirability bias. Theses situations can be differential because people with poor health can remember or attributed it to the ADLs incapacities.	Thank you for highlighting this. The self-reported measures in this analyses were self-reported health and difficulties undertaking activities of daily living/instrumental activities of daily living/mobility.<br><br>Self-rated health: We chose self-reported health as it is widely demonstrated to be a robust measure of health, across numerous studies. There is currently no strong evidence to suggest that over or under-estimation of one's health undermines the validity of this measure as an indicator of health.<br><br>Self-reported difficulties with ADLs/IADLs: we do not agree that self-reported difficulties with ADLs/IADLS can lead to under or overestimation, as the nature of experiencing difficulty is inherently subjective and can vary day to day. Evidence shows that performance-based measures of ADL/IADL disability may fail to capture some of the day-to-day variation in the difficulties people experience, a limitation that a self-rated measure overcomes.
3) Confounding bias: others factors like level of schooling, ethnicity, residency, social support accessibility or healthcare accessibility, environmental factors (weather, road, light, housing etc..) were not take in account	We would agree that there are many confounding variables that could influence the outcome (self-reported health). However, it was not feasible for us to account for all factors as this this would result in an overfitted model. Indeed, as we describe in the discussion, we tried adjusting for other variables but instability in estimates from model to model signified risk of overfitting. Our model therefore includes the most important factors, including a measure of socioeconomic status (wealth, which we would argue is a more sensitive measure of socioeconomic disadvantage in later life than schooling).

	In terms of environmental factors, these may be relevant in some countries, however, weather, roads and light levels have relatively low variability across England.
4) This is a cross sectional study, there isn't temporality between exposure and outcomes. We can't conclude that poor health was presence before or after the unmet financial managing support.	Yes, we would agree with this statement. There is a paragraph in the discussion about the cross sectional limitation of this work. We have appended this to note the issues of temporality:<br><br>Our analysis also made use of cross-sectional data. This approach may not be ideal to examine the potential impact of individual unmet care needs on health, especially where such impact may be manifest over time. Longitudinal studies of health trajectories and changing support for daily living may offer greater insights, as well as confirm temporality between unmet needs and poor health.
5) The small size of people exposed to unmet need for social support care can't also explained the large CI95% around the ORs so the imprecision of these estimates.	If we have understood your comment correctly, your argument is that the relatively small number of people reporting unmet need **cannot** account for the larger CIs. We would disagree with this: as we have noted in the discussion, we think this may **partly** explain the imprecision in the estimates.
Reviewer: 3<br>Dr. Christina Aggar, Southern Cross University	Thank you for your helpful comments. Our responses are summarised below.
It is an interesting paper, however the data was pre COVID and I think that this needs to be acknowledged. We now know that loneliness is one of the most important social care needs that impact healthy ageing.	Thank you – we agree this is an important point and we have added the following in the discussion:<br><br>The data also represent a pre-COVID population, and it is therefore possible that the link between unmet social care needs may look different given the prolonged social isolation experienced by older people during the pandemic.
Another question I would ask - who supported the unmet need/s, family or formal carers, as this is also known to impact healthy ageing. Otherwise the shortcomings of the current data, whilst many, have been addressed	In this work, we measured 'absolute' unmet needs, which identifies populations who report a difficulty, but receive no help, with an ADL/IADL/mobility limitation. On this assumption, our study population received no support for these needs.

VERSION 2 - REVIEW

Reviewer 2
Name Attisso, Eugene
Affiliation Quebec National Institute of Public Health
Date 09-Dec-2024
COI

I appreciate the answers of authors.

This is great study for England's elders.

Please consider in furthers studies to measure fraily which encompass several dimensions of social needs cares more than ADLs.
